# Supplementary material for: From sequence to enzyme mechanism using multi-label machine learning
Source: BMC Bioinformatics. 2014 May 19;15:150. doi: 10.1186/1471-2105-15-150 (PMC4229970; doi:10.1186/1471-2105-15-150)
Supplement: Additional file 2 — Java code of ml2db. Additional file ml2db_code.tar.gz contains the Java source code to run the multi-label machine learning experiments and save the results to database. The code’s Javadoc is included. [file 1471-2105-15-150-S2.zip › additional file 2/ml2db/ml2db/doc/mulan/classifier/meta/class-use/MultiLabelMetaLearner.html]

Uses of Class mulan.classifier.meta.MultiLabelMetaLearner


JavaScript is disabled on your browser.


- Overview
- Package
- Class
- Use
- Tree
- Deprecated
- Index
- Help

- Prev
- Next

- Frames
- No Frames

- All Classes

## Uses of Class mulan.classifier.meta.MultiLabelMetaLearner

- Packages that use MultiLabelMetaLearner

  | Package | Description |
  | --- | --- |
  |  |  |
  | --- | --- |
  | mulan.classifier.meta |  |
  | mulan.classifier.meta.thresholding |  |
- - ### Uses of MultiLabelMetaLearner in mulan.classifier.meta

    Subclasses of MultiLabelMetaLearner in mulan.classifier.meta

    | Modifier and Type | Class and Description |
    | --- | --- |
    |  |  |
    | --- | --- |
    | `class` | `ClusteringBased` Class implementing clustering-based multi-label classification. |
    | `class` | `EnsembleOfSubsetLearners` A class for gathering several different SubsetLearners into a composite ensemble model. |
    | `class` | `HMC` Class that implements a Hierarchical Multilabel classifier (HMC). |
    | `class` | `HMCNode` Class that implements a node for the `HMC` |
    | `class` | `HOMER` Class implementing the Hierarchy Of Multi-labEl leaRners algorithm. |
    | `class` | `RAkEL` Class implementing a generalized version of the RAkEL (RAndom k-labELsets) algorithm. |
    | `class` | `RAkELd` Class implementing a generalized version of the RAkEL-d (RAndom k-labELsets) algorithm with disjoint labelsets. |
    | `class` | `SubsetLearner` A class for learning a classifier according to disjoint label subsets: a multi-label learner (the Label Powerset by default) is applied to subsets with multiple labels and a single-label learner is applied to single label subsets. |
  - ### Uses of MultiLabelMetaLearner in mulan.classifier.meta.thresholding

    Subclasses of MultiLabelMetaLearner in mulan.classifier.meta.thresholding

    | Modifier and Type | Class and Description |
    | --- | --- |
    |  |  |
    | --- | --- |
    | `class` | `ExampleBasedFMeasureOptimizer` This class takes the marginal probabilities estimated for each label by a multi-label learner and transforms them into a bipartition which is approximately optimal for example-based FMeasure. |
    | `class` | `Meta` Base class for instance-based prediction of a bipartition from the labels' scores |
    | `class` | `MetaLabeler` Class implementing the MetaLabeler algorithm. |
    | `class` | `MLPTO` Class that implements the Multi Label Probabilistic Threshold Optimizer (MLTPTO). |
    | `class` | `OneThreshold` Class that estimates a single threshold for all labels and examples. |
    | `class` | `RCut` Classs that implements RCut(Rank-based cut). |
    | `class` | `SCut` Class that implements the SCut method (Score-based local optimization). |
    | `class` | `ThresholdPrediction` Class that learns to predict a different threshold per exampleFor more information, see |

- Overview
- Package
- Class
- Use
- Tree
- Deprecated
- Index
- Help

- Prev
- Next

- Frames
- No Frames

- All Classes
